# Pair interactions in online assessments of collaborative problem solving: case-based portraits

**DOI:** 10.1186/s41039-018-0079-7

**Published:** 2018-08-09

**Authors:** Johanna Pöysä-Tarhonen, Esther Care, Nafisa Awwal, Päivi Häkkinen

**Affiliations:** 1 Finnish Institute for Educational Research, University of Jyvaskyla, P.O. Box 35, 40014 Jyvaskyla, Finland; 2 Assessment, Curriculum and Technology Research Centre, Melbourne Graduate School of Education, The University of Melbourne, Melbourne, VIC 3010 Australia; 3 The Brookings Institution, 1775 Massachusetts Ave., NW, Washington, DC 20036 USA; 4 Assessment Research Centre, Melbourne Graduate School of Education, The University of Melbourne, Melbourne, VIC 3010 Australia

**Keywords:** Case studies, Collaborative problem-solving, Computer-supported collaborative learning, Directed content analysis, Small-group processes, Social aspects of learning and teaching, Qualitative research

## Abstract

This exploratory case study focuses on how pairs of students can build a shared understanding and acquire collaborative problem-solving (CPS) practices during an online assessment of CPS skills, which is seen in the context of the CPS construct, in a symmetrical and asymmetrical task type. Even though CPS is widely recognised as a core twenty-first-century competency, its nature is not yet well understood. Also, until recently, most of studies have focused on the individual’s solution to a problem or on the skills individuals bring into a problem-solving space. This study extends from an individual- to group-level focus in CPS, emphasising the role and quality of the social aspects in CPS processes and outcomes. Focusing on the group level because it mediates multiple levels of learning, including individual cognition and socio-cultural practices, may provide us with a better understanding of how pairs establish CPS practices. Because of the complexity of CPS and the general challenges of remote collaboration in an online context, the study relies on the triangulation of multiple data sources and phases of analysis. In this paper, the aim is to explore and visualise through contrasting case-based portraits of two pairs how micro-interaction processes evolve at the pair level. The results show that despite students’ similar CPS performance outcome scores and task designs aimed to facilitate collaboration, variations in micro-interactions occur across pairs, for example as individual and joint solution endeavours and as balanced and unbalanced dynamics of group interactions. Studying these patterns at the pair level may provide new insights into CPS and support strategies for acquiring these practices.

## Introduction

The current article focuses on the role and quality of the social aspects in collaborative problem-solving (CPS) processes and outcomes, which is explored during an online assessment of CPS skills in pairs of university students in a remote collaboration context. Recently, CPS has received increasing interest as one of the most important generic skills to be mastered, as illustrated by large-scale national and international assessments (e.g. Assessment and Teaching of 21st Century Skills (ATC21S) project, www.atc21s.org, and OECD’s PISA 2015 study, http://www.oecd.org/pisa/; see also Graesser et al. 2017). CPS and other generic skills, or so-called twenty-first-century skills, such as communication, creativity, critical thinking, digital literacies and more, are not seen as particularly new but are valued as critical for future learners and workers (e.g. Care et al. 2018; Care et al. 2016; Graesser et al. 2017; Griffin and Care 2015; Griffin et al. 2012; Scoular et al. 2017). Problem-solving skills, for example, have been assessed for several decades, for instance, in the domain of mathematics, but in current studies and policy papers, these skills are considered discipline-free skills that should be mastered as the actual focus of learning (Harding et al. 2017).

Although slightly different frameworks of CPS exist, the specific skills and expectations viewed necessary for the new workforce are consistent across the various frameworks. In the current study, the definition of CPS is consistent with that of Hesse et al. (2015). In the present study, our theoretical understanding of CPS builds on the ATC21S project and its extensive framework for technology-enhanced formative assessment of CPS skills (Hesse et al. 2015; Scoular et al. 2017). In this regard, Hesse et al. conceptualised CPS as a complex skill that links critical thinking, problem solving, decision making and collaboration across both social and cognitive domains. CPS is deemed more complex than merely working together and requires people working together on the same task to pursue a common goal where division of labour exists but where input is interwoven. In CPS, the quality of interactions distinguishes good collaboration (Dillenbourg 1999). In this context, collaboration requires active participation through searches for relevant information, joint use of resources, shared evaluations and an agreement on strategies and solution paths (Care and Griffin 2014). In CPS, individuals may possess different skills and information, but they must work collaboratively and exchange ideas to maximise their joint outcome toward a shared goal. The skills defined by CPS are pertinent to solving problems that are, by definition, complex, ill-structured and ambiguous.

**Fig. 1:**
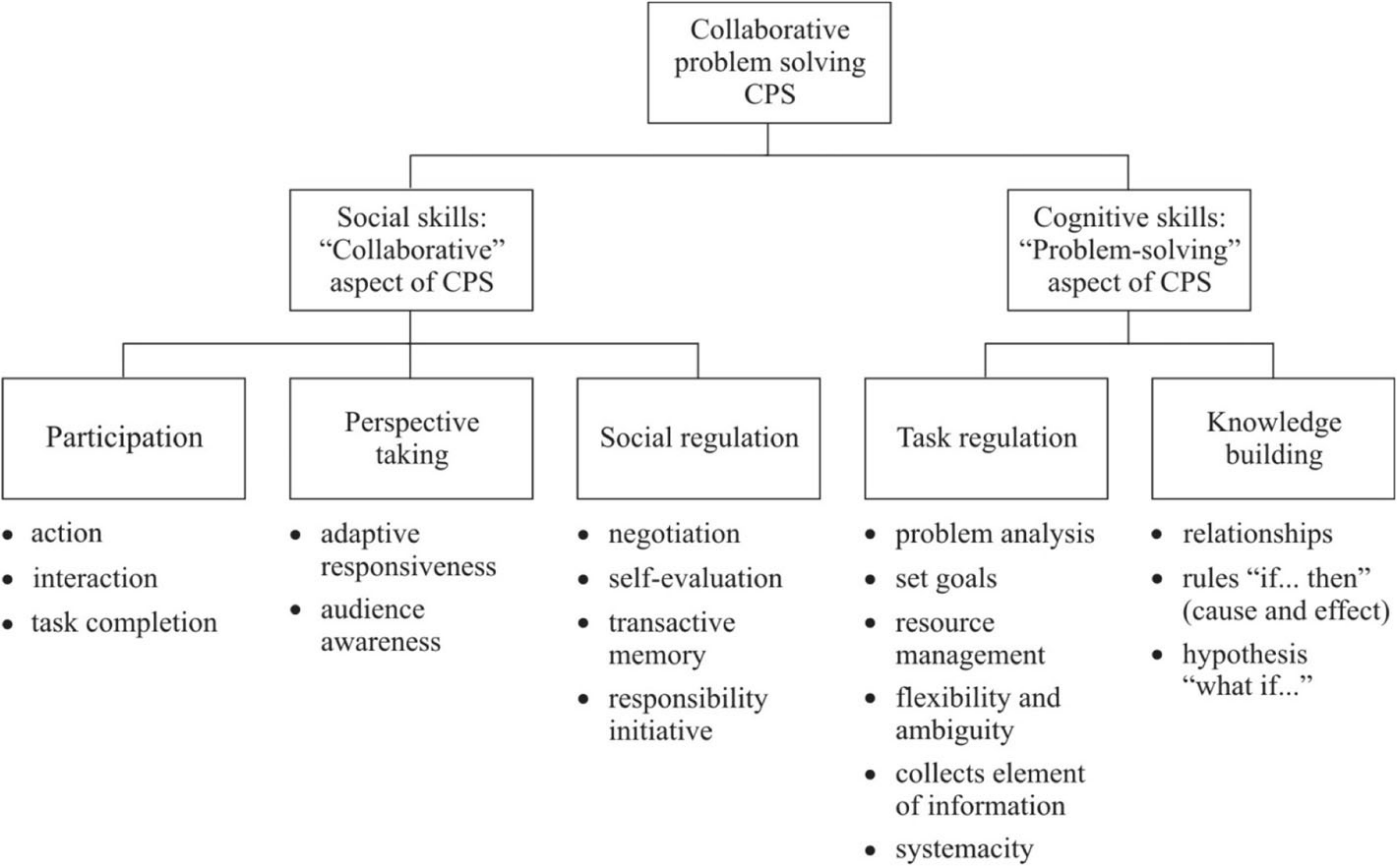
The set of CPS skills and subskills from the ATC21S CPS framework (modified from Hesse et al. 2015; see also Care et al. 2016; Scoular et al. 2017)

Accordingly, CPS comprises a set of subskills that consist of five strands of individual- and group-level capacities under the broad social and cognitive skills (Hesse et al. 2015). The two hypothesised components of CPS are not mutually exclusive. The social component draws on the literature from social and organisational psychology while the cognitive component draws heavily on classical approaches to individual problem solving. Following Hesse et al. (2015), social skills (i.e. participation, perspective taking and social regulation) are about managing participants (including oneself), which refers to the ‘collaborative’ part of this method. Cognitive skills (i.e. task regulation and knowledge building) are about managing the task, which refers to the ‘problem solving’ part of the method. From the social strands of the CPS construct, ‘participation’ refers to the readiness to share information and externalise thoughts, ‘perspective taking’ means the ability to take into account others’ perspectives and ‘social regulation’ points to the awareness of the strengths and the weaknesses of the group members (i.e. group/team awareness; see Fransen et al. 2011). From the cognitive strands of the CPS construct, ‘task regulation’ is defined as planning and monitoring the skills for developing strategies for problem solving and shared problem representation (i.e. ‘joint problem space’, see Barron 2003; Roschelle and Teasley 1995), whereas ‘knowledge building’ here refers to the ability to learn and build knowledge through group interaction. Within each of these skills, there are subskills (together, 19 subskills, including the ‘solution’), which are demonstrated by observable indicators of actions or processes (for an overview of the ATC21S CPS framework, see Fig. 1).

In the ATC21S project, a technology-enhanced assessment environment was designed, comprising a series of Web-based interactive, game-like tasks (see e.g. Care et al. 2015). The tasks were designed to measure the skills, subskills and processes characterised by the CPS construct during dyads participating in the CPS activities. The ATC21S assessment tasks, discussed later in the present paper, rely on and capture these actions or processes to measure and reflect the CPS construct, as defined by Hesse et al. (2015).

Although CPS is widely recognised as a core competency for twenty-first-century learning, its nature is not well understood particularly in terms of its development trajectory from basic to more sophisticated levels (Scoular et al. 2017). Also, in the ATC21S assessment environment, the primary focus has been on how to assess the skill of the individual student in collaborative partnership (Care et al. 2016). In this exploratory case study, the focus is extended to group-relational aspects of CPS (Pöysä-Tarhonen et al. 2016; Pöysä-Tarhonen et al. 2017). The current study emphasises the *role* and *quality* of the social aspects of CPS processes and outcomes (e.g. Barron 2003; Dillenbourg et al. 2016; Simpson et al. 2017; Wegerif et al. 2017). Accordingly, one part of CPS is the task, and the other part is the environment within which the participants create and share knowledge, monitor their progress and detect and repair the breakdowns in communicative acts that may hamper collaboration (Alterman and Harsch 2017; Roschelle and Teasley 1995). The current study concentrates on the second part of CPS, which is seen vital in computer-supported collaborative learning (CSCL) research, as it is the context of the current study. In earlier work, it has been found, for example, that in collaborative learning and problem solving, the processes of building a shared understanding of the situation at hand and the quality of interactions appeared to be, to some point, even more important than the actual abilities of the individual participants when it came to solving the tasks (Dillenbourg et al. 2016; see also Barron 2003; Simpson et al. 2017).

The current study takes a small-group focus on CPS practices. Studies on collaborative learning, in general, have reduced their scope to study either individual cognition or socio-cultural practices (Stahl 2017). As Stahl (2017) stated (in learning research), ‘Traditional methods (pointing to quantitative or qualitative methods that analyse net changes) often provide evidence *that* change has taken place, but do not describe *how* the change has taken place’ (p. 114, original emphasis). Following Stahl (2017), it is argued that an emphasis on small-group practices, because it mediates the multiple levels involved in learning (e.g. individual cognition on the one hand and the socio-cultural practices of the classroom and tools used on the other), may provide us with a foundation to better understand how CPS takes place in this context. Notably in the current study, from theoretical and methodological points of view, we regard pairs as small groups (Williams 2010). As Williams (2010) stated, despite certain properties that do not lend themselves to small groups (i.e. coalition formation or the difficulty to imagine newcomers and old-timers in a group of two), ‘[i]n most instances dyads are groups of two and operate under the same principles and theories that explain group processes for groups of three and larger’ (p. 268).

To explore CPS as a dynamic, group-level set of processes in an online setting, a case-based study was undertaken. As Yin (2009) argued, the *how* and *why* questions are better answered through a case-based approach because they are well suited for exploring complex social phenomena. In case study research, an intensive study of a single unit can provide ways to better understand a larger class of similar units, especially when relying on the triangulation of multiple data sources (Baškarada 2014; Gerring 2004; Hmelo-Silver et al. 2009; Yin 2009). Therefore, in the current study, the data comprise objective measures and process data (i.e. autoscoring of CPS skills, activity logs and screen recordings) combined with subjective process data (i.e. cued retrospective reporting (CRR) interview data) to form the basis for the descriptive analysis of pair-level CPS processes, which are actualised as process visualisations of micro-interaction processes (see Davis et al. 2015). In the majority of the ATC21S studies to date (see Care et al. 2018), the scope of analysing the social aspects has been on the placement (and occurrence) of chat actions in the CPS process (Care et al. 2015) rather than the chat content itself.

Focusing on the ‘how’ of CPS requires an analysis that goes beyond performance scores or the solution to the problem. The ‘how’ explores whether different sets of interactions can underlie successful completion and whether the nature of a task stimulates different sets of interactions. For example, two styles of tasks have been used to assess CPS (Care and Griffin 2017). One style presents and gives access to the same stimulus set for both the students in a pair, and the other presents and gives access to different stimulus sets for the two students. The first style is typically referred to as symmetric and the second as asymmetric. According to Care et al. (2015), the accessibility here refers to ‘both to direct retrieval as well as human capacity to understand and manipulate the required artefacts- be these object, knowledge, or processes’ (p. 87). This difference between the task types can reasonably be hypothesised as stimulating different sets of skills that can be applied to the task. In a symmetric task type, collaboration may be valued for its social aspect but may also be regarded as a counter to the students’ interests (Care et al. 2015). In turn, the situation of accomplishing an asymmetric task, where resources are not equally accessible to both students in the pair, is expected to increase the need for collaboration or better prompt collaboration and the collective activity to develop (Care et al. 2015).

Taken together, in the current study, we have broadened the analysis beyond the measured CPS skill levels of the individual students, having them search for the differences in the *quality of CPS processes at the pair level* during the online assessment of CPS skills, in regard to the different types of the tasks. Based on multiple, rich data and multiple phases of analysis, we ask the following:

1. Are there differences in the quality of the micro-interactions at the pair level that are underlying similar CPS measures of individual students?
2. Does the task type (i.e. symmetric/asymmetric) qualitatively provoke different micro-interactions in the student pairs?

## Methods

### Participants and tasks

The research participants (*n* = 20) were students enrolled in a master-level teacher education programme at a Finnish university. Students were randomly assigned to their pairs. Each student pair completed one bundle of tasks in the ATC21S assessment environment. The tasks were developed at the Assessment Research Centre at the University of Melbourne from 2010 to 2013. The assessment environment is based on human-to-human approaches for assessing CPS (Care et al. 2015) and comprises a set of online interactive and collaborative problem-solving tasks in STEM domains. In the tasks, the student pairs are given a unique subset of resources that are required to solve the problem. To fully understand the problem space and identify all the necessary resources, the students need to rely on their partner (Care et al. 2015; Care et al. 2016). The communication takes place via a free-form chat interface.

The set of tasks used in the current study comprised four diverse tasks, together lasting approximately 60–90 min. During the tasks, student pairs (students A and B) were seated in different classrooms to ensure that the only means of communication was the chat interface. For this study, the bundle comprised ‘Laughing Clowns’ and ‘Olive Oil’, which are content-free tasks, and ‘Plant Growth’ and ‘Small Pyramids’, which are content-dependent tasks (see Care et al. 2015). Content-free tasks enhance inductive and deductive thinking whereas content-depended tasks require students to apply knowledge related to curriculum.

**Fig. 2:**
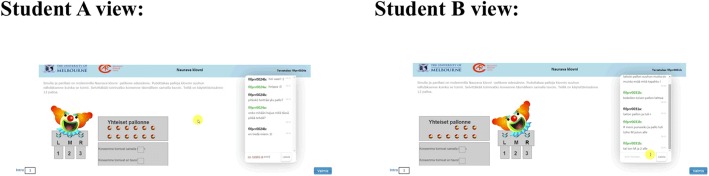
A screen capture from the screen activity data (perspectives of students A and B, Laughing Clowns) (In Finnish)

**Fig. 3:**
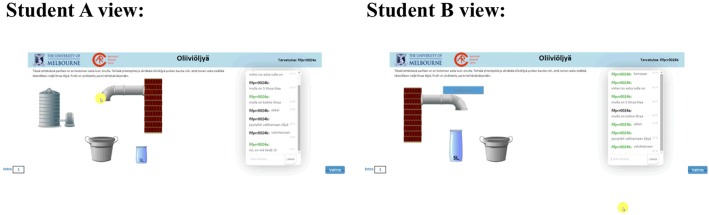
A screen capture from the screen activity data (perspectives of students A and B, Olive Oil) (In Finnish)

Laughing Clowns is a symmetric task, whereas the other three are designed as asymmetric. According to Care et al. (2015), in a symmetric task, the student pair is presented with the same images, perspectives, instructions and resources within the online task space. In an asymmetric task, the student pair is presented with asymmetrical perspectives, providing different information and resources (for similar and different views of the pairs in a symmetric and asymmetric task, respectively, see Figs. 2 and 3). In the current paper, we explore the micro-interaction processes of pairs in comprising (a) a symmetric task (Laughing Clowns) and (b) an asymmetric task (Olive Oil). These tasks are content-free tasks that do not require any prerequisite knowledge of the curriculum and merely rely on the application of reasoning (see Care et al. 2015). We also reduced the scope on the content-free tasks of the bundle used in this study. This characteristic may enhance comparability of the individuals, since differential access to the prior knowledge is rendered irrelevant.

In the Laughing Clowns task, two participants are presented with a clown machine and 12 balls to be shared between them. The goal is for the students to determine whether their clown machines work in the same way. To do this, the two students need to share information and discuss the rules, negotiating how many balls they should each use. The students must place the balls into their clown’s mouth while the mouth is moving to determine the rule governing the direction the balls will go (entry: left, middle and right; exit: positions 1, 2 and 3). Each student must then indicate whether they believe the two machines work in the same way. Students do not have access to each other’s screen. In Fig. 2, the views of students A and B are shown.

In the Olive Oil task, participants are presented with different views of the task, including different resources. The goal is to fill a jar with 4 l of olive oil with the available resources. To achieve this objective, the students must work out what resources are available and how to sequence the use of these resources. Student A has a virtual 3-l jar, olive oil dispenser, transfer pipe and bucket. Student B has a virtual 5-l jar, transfer pipe, accept button and bucket. None of the participants are aware before beginning communication what is available to their partner. The pair thus needs to recognise that student A must fill his or her jar at the dispenser and place it under the transfer pipe so that student B can accept the oil transferred by his or her partner from the pipe. Until this point, student B cannot complete any meaningful actions and is dependent on the actions and interactions on student A. The students need to explore and navigate the task space together until they manage to place 4 l of oil in student B’s jar. This task follows the reasoning processes required in the Tower of Hanoi problem, which was popularised by mathematician Eduard Lucas in 1883 (Newell and Simon 1972; Petković 2009). In the Olive Oil task, both problem solvers are required to work out a sequence of movements to achieve the goal of the task. Students still do not have access to each other’s screens, and the task is complicated by the division of resources and dispersed information among dyads. Figure 3 shows the different perspectives of students A and B.

### Data collection

#### Objective measures and process data: auto-scoring and screen recordings

In the ATC21S assessment environment, students are assessed individually during dyad participation in CPS activities. CPS tasks are designed to generate specific behaviours, aligned with the skills and subskills of the CPS construct (see Fig. 1; Care et al. 2016; Hesse et al. 2015; Scoular et al. 2017). Students’ completion of the tasks generates log stream data (i.e. the activity log), and the patterns in these data (i.e. the movement of artefacts and the occurrence of chat) are captured algorithmically and are coded by the scoring engine and then scored according to the Rasch model, producing information on students’ social and cognitive skill levels (see Adams et al. 2015; Care et al. 2016). The activity log shows the events between two students working on the task. (For an example of activity log, see Table 1).

**Table 1:** A section from an activity log (task Laughing Clowns) (translated from Finnish)

Task	Page	Player	Event type	Contents	Timestamps
23	1	A	Chat	Do you have any clue what we’re supposed to do here?	06/10/2015 16.45
23	1	B	Chat	I don’t know I’m thinking :D	06/10/2015 16.45
23	1	A	Chat	Yeah, would you throw first?	06/10/2015 16.45
23	1	B	Chat	Let’s throw a ball and see what happens	06/10/2015 16.45
23	1	B	Action	startDrag:ball1:70:150	06/10/2015 16.45
23	1	B	Action	stopDrag:ball1:70:150	06/10/2015 16.45
23	1	B	Action	startDrag:ball1:70:150	06/10/2015 16.45
23	1	B	Action	stopDrag:ball1:509:135	06/10/2015 16.45
23	1	B	Action	dropShuteL:ball1:509:135	06/10/2015 16.45
23	1	B	Chat	Where did it come out for you?	06/10/2015 16.46

Table 1 displays the task and player IDs (task 23, student A or B), the page of the task (here, page 1), actions (e.g. the event type), the contents of the chat (text exchanged) and their timestamps

Accordingly, to capture the CPS skills and the subskills shown in Fig. 1, indicative behaviours are proposed to suggest the evidence of the behaviour being observed, demonstrating the presence or absence of a particular subskill and how much of that subskill is present in an individual (see e.g. Adams et al. 2015; Care et al. 2016). Examples of the CPS skills and the indicative behaviours in the Laughing Clowns and Olive Oil tasks are shown in Table 2.

**Table 2:** Example of social or cognitive skills and indicative behaviours for exemplar tasks in Laughing Clowns and Olive Oil (adapted from Care et al. 2015)

CPS elements/subskills	Indicative behaviour	Evidence of data	
		Laughing Clowns	Olive Oil
Interaction	Interacts with the partner	Presence of chat before allowing the partner to make a move	Presence of chat before allowing the partner to make a move
Audience awareness	Adapts contributions to increase understanding for the partner	Number of ball moves attempted before stopping and waiting for the partner to move or respond	Presence of info exchange on individual container states
Resource management	Manages resources	Realises that balls are meant to be shared and uses only allotted half	(Not observed)
Relationships	Identifies connections and patterns between elements of knowledge	The two students come to an agreement on how their machine works	Presence of chat exchanging information when A or B recognises the significance of his or her jar containing only 1 l
Reflects and monitors	Adapts reasoning or course of action as information or circumstances change	(Not observed)	Learning from redundant activities, such as A moving jar to bucket
Solution	Arrives at the correct answer	Identifies connections and patterns between elements of knowledge	Last action requires B’s jar to contain 4 l of oil

Behaviours hypothesised as indicative of particular subskills or elements are thus extracted from the log stream through automated coding processes. Auto-scoring captures two types of response representing common and unique events (Adams et al. 2015; Care et al. 2016). Common events include beginning and end of a task, system messages confirming actions and providing navigation, and chat between partners. Unique, or local, events include those that are specific to task. For the Laughing Clowns task, for example, these include the drag and drop of a ball and its location. A count of the ‘dropShute’ actions (dropping the balls into the clown’s mouth, see Table 1), for example, can be coded to indicate how well a student managed their resources (the balls). These coded indicators are classified as either direct or inferred. Indicators that are classified as direct are those that describe a particular action, which can be interpreted in its own right. Indicators classified as inferred include those which include a sequence of actions, from which sequence a particular thinking or exchange process can be inferred. For example, if one student takes a particular action, there is then a chat exchange, followed by the student changing the action, it can be inferred that the chat of the partner has influenced the first student. An example of the auto-scoring approach is provided in Table 3.

**Table 3:** Use of algorithm to relate actions to the element *systematicity* of the collaborative problem-solving construct (adapted from Adams et al. 2015)

Indicator name	Details	Algorithm	Output
U2L004A U2L004B	Systematic approach All positions have been covered. Scoring rule: threshold value Task name: Laughing Clowns	Step 1: Find all drop ball occurrences captured as dropShute and their corresponding positions as dropShuteL, dropShuteR and dropShuteM. Step 2: Then count all the occurrences of the action recorded under ‘dropShute’ and their unique positions from the log. Step 3: Increase the value of the indicator by 1 if 1 or more ‘dropShute’ occurs in the form of dropShuteR, dropShuteL or dropShuteM. Step 4: If the total number of unique dropShutes (dropShuteR, dropShuteL and dropShuteM) from the log is less than 3, the value of the indicator is defined as − 1 to indicate missing data	Count values

In Table 3, the first column represents the name of the indicator, being unique for each indicator. In the example, ‘U2’ points to the Laughing Clowns task, ‘L’ refers to local and explains that the indicator is unique to the task, ‘004’ means that the indicator is fourth created for the task and ‘A’ means that the indicator is applicable to students A and ‘B’ that it is applicable to student B

As described in Adams et al. (2015), data are converted into dichotomies or partial credit, with each indicator being treated somewhat similarly to items on a traditional test. In turn, these are aggregated with like indicators across set bundles of tasks. Cut-off values to determine levels of quality across subskills are calculated for the purpose of scaling. Applying the Rasch measurement model, it is possible to determine the patterns within the CPS conceptual framework and to interpret a student’s CPS ability by estimating the probability of success on a behavioural indicator (equivalent to a test item) given the relative position of an individual and item to an underlying construct (Adams et al. 2015). Thus, we can infer an individual’s skills from his or her activities done during the task in the same way that we can infer an individual’s content knowledge from his or her responses in traditional test questions.

In addition, CamStudio™ software (http://camstudio.org) was used for recording all screen activities during the CPS sessions. In the beginning of the session, the students were informed of the recording intention and given detailed instructions on how to start recording their portal session and save their data. During the portal session, the students were also requested to check whether the programme was running accurately.

#### Subjective measures: cued retrospective reporting

To obtain subjective measures on the CPS processes, the CRR (e.g. van Gog et al. 2005) was applied to the problem-solving tasks. CRR is defined as a verbal reporting procedure in which, based on a cue or cues of their performance, participants are invited to verbalise their thought processes during the task performance retrospectively (van Gog et al. 2005), also previously applied in problem-solving tasks (van Gog and Scheiter 2010). In the current study, through verbal reporting, the aim was to make the CPS process explicit through students’ self-monitoring of why and how they took the actions, especially as a student pair. The CRR interview was cued with the recorded screen activity data (i.e. screen capture videos), including the mouse operations and chat discussions recorded during the assessment session. During the interview, the researcher only prompted the interviewee to verbalise or continue if she or he seemed to pause for a longer time. The CRR sessions were videotaped. During the interview, the video camera was directed at the computer screen (showing the screen capture video recorded during the CPS session) to capture the exact point of time discussed. These were also noted in the transcriptions of the interviews (for an example of CRR data, see Table 4).

**Table 4:** Example of verbal reports (CRR data, Laughing Clowns) (translated from Finnish)

	Task 1: Laughing Clowns
Student A	Well hmm, here, in this exercise I realised at once what it is all about. Uh, maybe I was a bit hasty in the sense that I didn’t read fully this task instruction, that we have some balls in common, but I sort of realised how my computer was functioning. Like one can see it from this, I realised like at once what the task there is. And then I just tried it out a few times how the thing was functioning and reported [gives a laugh] right away. I don’t know whether one could have like [a short laugh] built some cooperation there like so that one would first have like told the instruction in the way one understood it. But well, on the other hand, as I knew who my partner was, I know that [the partner] too is really smart and good at these, so... and then, I just (thought) that she’s likely to get it from there quite quickly as well. This was quite a good warm-up exercise all right, and so. I don’t know if I got the answer right, but I think I did. [pause] and [the partner] might well write this onto chat a bit more smartly, however, how that computer of hers was functioning. As I wrote it in text format like that, so she wrote it this way more elegantly [by using letters and numbers appearing on the clown figure]. Whatever way she wrote it then. At that point I was a little afraid that did she realise that the balls ran out. So then she wrote it this way, a bit more smartly. 03:13; 03:25 (I’m) slightly impatient in these tasks, so well [laughing] it may happen that I keep clicking the Finish button a few times or something like that. R: This was all. (-) yes. S: Yes. I had already answered and [the video shows that Student A has already finished but is still waiting for his partner] then you may also need to give a slight hint that should we move on [giving a laugh]. I’m used to do a lot of this kind of problem-solving tasks, but I’ve always done them alone, so this was a challenge in that sense.
Student B	Well my partner (didn’t realise perhaps) that I had checked it. Then there at one point I still tested and then I wrote down where they go, so that if I drop there from L. And then I tried to test the same ones like in those if it goes always like in the same way. But then at some point when those partner’s messages appeared there so then I still tried with those, and then did it at the same time (you know), and then I wrote it down like that [as letters and numbers in chat] so that where they go from those. Because if I just had looked how they go (--) if I drop from L, for instance, so that it will go to number one, so I would not necessarily have remembered it then. And then I wrote it there already, (--), like it will probably go. Yes, then I like, there at the point when I was checking those, so my partner (-) and I realised that the way it is (thought), this R, and then this, this middle one. so then just as that one didn’t go straight, so I like realised it at that point [that the computers were functioning differently]. Then I like confirmed what the outcome was. 05:16; 05:25 Then I was you know [gives a laugh] (--). Or this partner of mine was quicker to write [laughs] than me, Then at times just - R: [asks something, not audible on tape] 05:50 S: Er, well perhaps not in this exercise, but in later exercises. Then he [the partner] solved them more quickly while, before I managed to solve them, so in the sense that of course I knew that I too was able to solve them and knew how to solve them, but I was still thinking. Like so that I already knew in my mind how it goes, but I hadn’t yet had the time to write it. So then my partner like put how it goes, then I like read the instruction, then I was you know like [nodding] I just hadn’t like written it yet.

### Data analysis

**Fig. 4:**
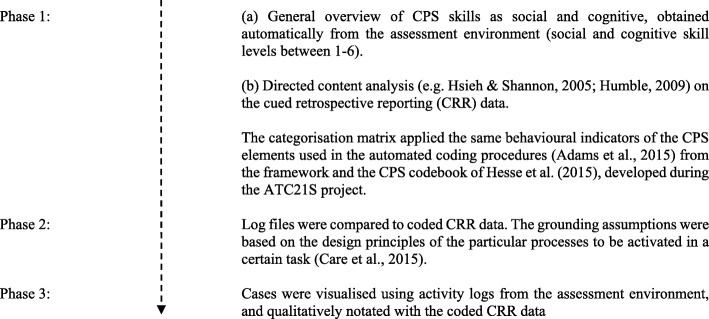
An overview of the multiple phases of data analysis

To better understand how CPS processes unfold at the pair level as micro-interactions (Davis et al. 2015), a triangulation analysis technique was used (Humble 2009; Meadows and Morse 2001). The analysis comprised three phases and multiple data sources (for an overview of the data analysis process, see Fig. 4). The analysis relied on the CPS performance measures, which were obtained from the assessment environment (phase 1a), combined with directed content analysis on the process tracing data (i.e. CRR data; phase 1b; Pöysä-Tarhonen et al. 2016; Pöysä-Tarhonen et al. 2017). In phase 2, to determine micro-interactions pertaining to CPS processes at the pair level and also to confirm the findings of the cumulative data accounts that were the result of the directed content analysis on CRR data, the log files were compared with the coded CRR data and analysed for congruence. Finally, in phase 3, selected cases were visualised using activity logs from the assessment environment and qualitatively notated with the coded CRR data.

#### Autoscored CPS skill levels

As discussed earlier, the ATC21S assessment environment automatically codes activity logs for individual student performance measures as social and cognitive skills (skill levels between 1 and 6; see Adams et al. 2015). These data were used for acquiring a general overview of the CPS skill levels of the participating students. At this point, four pairs (eight students) were selected for further analysis based on equal technical quality and availability of all the data (including activity logs from the assessment environment, screen recordings and CRR data).

#### Directed content analysis

CRR interviews resulted in qualitative retrospective reports of the social and cognitive aspects of CPS processes from the perspective of an individual student (see Table 4). A directed content analysis that included the application of conceptual categories to a new context (Hsieh and Shannon 2005; Humble 2009) was applied to the transcribed CRR data. The categorisation matrix of these data applied the same task-specific behavioural indicators of the CPS elements (skills and subskills) as defined by Hesse et al. (2015) that was used in the automated coding procedures in the assessment environment (Adams et al. 2015). The unit of analysis was an episode or passage: a minimum unit, where a certain criterion of the pre-determined category of a particular CPS element was observed at the pair level. In Tables 5 and 6, the categorisation matrices for the Laughing Clowns and Olive Oil tasks, respectively, are presented (for more examples, see Care et al. 2015).

**Table 5:** The categorisation matrix of the behavioural indicators of CPS elements in the Laughing Clowns task

Skill/element	Behaviour
Social	
Interaction	Interacting with partner
Audience awareness	Adapts contributions to increase understanding of others
Responsibility initiative	Takes responsibility for progress for the group
Cognitive	
Resource management	Manages resources
Systematicity	Implements possible solutions to a problem
Relationship	Identifies connections and patterns between elements of knowledge
Solution	Correct answer

**Table 6:** The categorisation matrix of the behavioural indicators of CPS elements in the Olive Oil task

Skill/element	Behaviour
Social	
Interaction	Interacting with partner
Cognitive	
Problem analysis	Identifies the necessary sequence of subtasks
Relationship	Identifies connections and patterns between elements of knowledge
Rules: ‘If…then’ (cause and effect)	Identifies a sequence of cause and effect
Reflects and monitors	Adapts reasoning or course of action as information or circumstances change
Solution	Correct answer

The combination of the CPS elements and their behavioural indicators (representing 19 subelements) are task specific and based on the different characteristics of the tasks (Care et al. 2015). Not all the elements are present in the different tasks. The criteria for the content analysis were ‘social’ and ‘cognitive’ as the main categories and the task-specific subelements of social and cognitive as subcategories. If the content was related to CPS but could not be connected to any of the CPS elements in the predetermined coding category, it was placed in a residual category named ‘unclassified’. The parts of the transcribed CRR data where no criteria could be found were left uncoded. In the current paper, the residual category was not included in the process visualisations. (Examples of the coded primary data from the cued retrospective reporting (CRR) data (from the Laughing Clowns task) are presented in Table 9 in Appendix 1).

To ensure the trustworthiness of the deductive approach applied in the current study, a double-coding procedure was used (Schreier 2012). Because the code definitions are clear, subcategories do not overlap (Adams et al. 2015). Two rounds of coding by the first author produced approximately the same results, which indicate a good quality of the deductive categorisation matrix (Schreier 2012). Also, to ensure sound interpretation of the data, the coding of the first task of the two pairs was verified by a co-author. For the directed content analysis, Atlas.ti^®^ data analysis software was used. Next, the coded data were exported as an xml file to Microsoft® Excel for organising, analysing and visualising. Based on the categorised data, cumulative frequency distributions were calculated to summarise the appearance of coded CPS elements by individual students across four different CPS tasks, which comprised the assessment bundle. Next, relative frequencies were calculated in pairs across different tasks.

#### Process visualisations of the micro-interaction episodes

As outlined earlier, evidence of collaboration between the students was derived by combining the activity logs (observed behaviour of CPS) with students’ interpretations (CRR data) of CPS processes in pairs (experienced CPS process). Following Davis et al. (2015), the dyadic interactions were not treated as a single interaction thread, but rather, they were seen as accumulation of multiple periods of interactions of various lengths of time (Davis et al. 2015). When searching for these pair-level ‘patterns’, we did not only focus on the content of the utterances but also looked for the connections across these utterances (see e.g. Sibert-Evenstone et al. 2016; Williamson Schaeffer 2017). Accordingly, in the fine-grained analysis of the contents of the CPS processes in the student pairs, to search for and determine the beginnings and endings of these micro-interactions within a student pair (see Davis et al. 2015), the grounding assumptions were based on the design principles of the particular task-specific CPS actions to be triggered in a certain task (Care et al. 2015). This means that we needed to define what connections were meaningful in terms of the CPS construct and, in the visualisations, to highlight the sequential meaning making at the pair level (Stahl 2017). Finally, in the visualisations, these meaningful CPS processes were notated with coded CRR data.

## Results

### How do collaborative problem-solving elements appear in different tasks (individual-level cumulative accounts)?

The CPS performance measures of all research participants, which were based on automated scoring of the assessment environment, did not show strong differences between the individual students. This was particularly the case for the social aspects (social skills were between 5 and 6, cognitive skills between 3 and 6); therefore, there was no clear distinction between ‘successful’ or ‘less successful’ pairs.

**Fig. 5:**
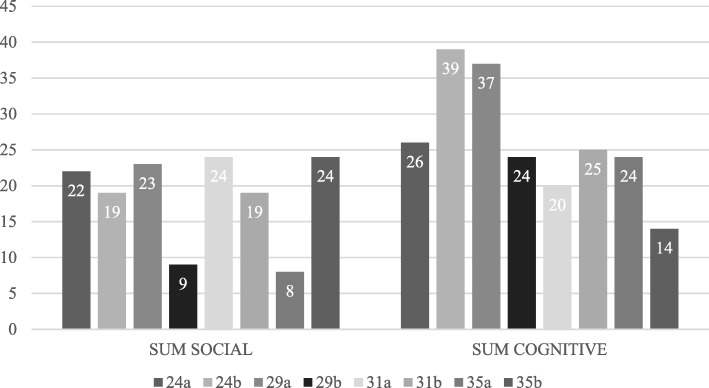
The sums of the frequency distributions of the CPS elements in the four tasks. The sums of the frequency distributions of the CPS elements in all the four tasks are presented at the level of an individual participant. The participants are displayed by their student IDs, such as students 24a, 24b and so forth, starting from the left side of the chart. For example, for student 24a, the sum of ‘Social’ is 22 and the sum of ‘Cognitive’ is 26, whereas for student 24b, the sum of ‘Social’ is 19 and the sum of ‘Cognitive’ is 39

The directed content analysis of CRR data resulted in a frequency distribution of CPS elements of the individuals across tasks. The sums of the social and cognitive elements of the individual students in all four tasks are presented in Fig. 5. These cumulative ratios show how students interpreted the problem-solving process. As visible in Fig. 5, six students (24a, 24b, 29a, 29b, 31b and 35a) of the eight highlighted more cognitive aspects in their CRR reports across the four tasks, whereas only two students (31a and 35b) highlighted more social aspects.

### Micro-interactions in pairs: contrasting case-based portraits of two pairs

Based on the directed content analysis combined with the observed behaviour over the CPS processes, contrasting cases of two pairs (pairs 24ab and 29ab) were chosen for visualisations of the qualitatively diverse micro-interaction processes of pairs across (a) a symmetric task (Laughing Clowns) and (b) an asymmetric task (Olive Oil). The skill levels across the pairs showed only a slight difference: pair 29 had lower social skill levels (student A: social 5, cognitive 4; student B: social 5, cognitive 3), and pair 24 had higher social skill levels (student A: social 6, cognitive 5; student B: social 6, cognitive 3).

The directed content analysis of the Laughing Clowns task revealed a lack of social aspects in the interpretations of pair 29 when completing the task, whereas for pair 24, the social and cognitive aspects were balanced and aligned with the original design of the task (see Table 7 for the relative frequencies of the social and cognitive aspects in pairs). In the original task design, the social aspects form 43% and cognitive aspects 57% of the elements assessed here. For the Olive Oil task, the directed content analysis did not show strong differences between the two pairs (see Table 8). In the original design of the task, the cognitive aspects are central (83.3%), whereas the social aspects form only 16.7% of the elements to be assessed in the task. If compared with the task design, both pairs 24 and 29 highlighted more social aspects in the CRR interviews [pair 24 (28.6%) and pair 29 (31.6%)]. However, the importance of collaboration is apparent in the task: the cognitive aspects are measured in accordance with the communicative acts between the pairs. Thus, the successful completion of the task would require joint efforts of the pair during particular processes of problem solving.

**Table 7:** Relative frequency distributions of social and cognitive aspects (Laughing Clowns)

Laughing Clowns	Social aspects (%)	Cognitive aspects (%)
CPS elements (original task design)	43	57
Student interpretations (based on CRR data)		
Pair 24	50	50
Pair 29	0	100

Relative frequency distributions of the social and cognitive aspects that are acquired from the CRR interviews (pairs 24 and 29) and seen in relation to the designed task elements of Laughing Clowns

**Table 8:** Relative frequency distributions of the social and cognitive aspects (Olive Oil)

Olive Oil	Social aspects (%)	Cognitive aspects (%)
CPS elements (original task design)	16.7	83.3
Student interpretations (based on CRR data)		
Pair 24	28.6	71.4
Pair 29	31.6	68.4

Relative frequency distributions of the social and cognitive aspects that are acquired from CRR interviews (pairs 24 and 29) and seen in relation to the designed task elements of Olive Oil

#### Case 1: joint solution endeavour versus individual solution endeavour in CPS observed in the symmetric task Laughing Clowns

**Fig. 6:**
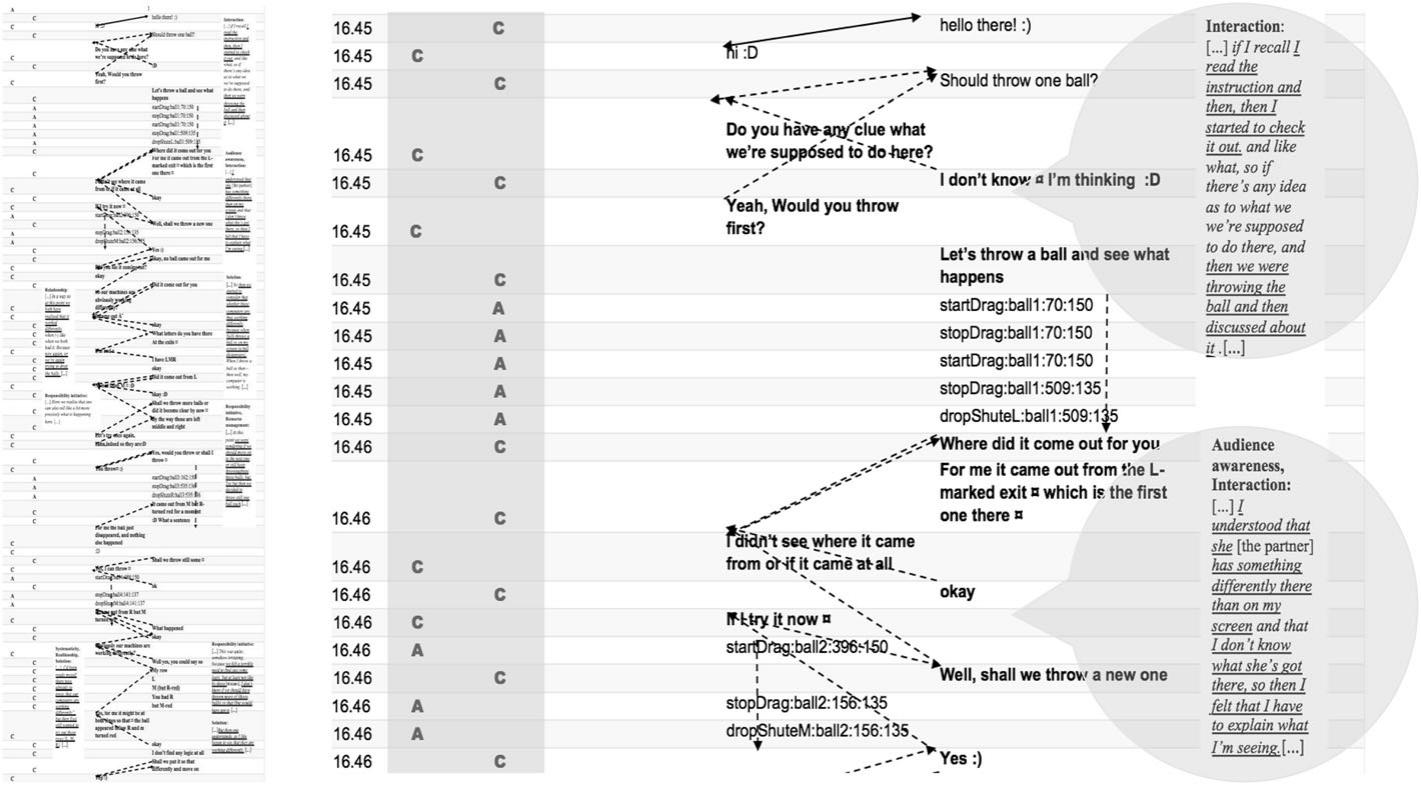
Joint solution endeavour (pair 24, Laughing Clowns). The full episode as a general pattern of interaction is presented on the left. On the right, an excerpt (indicated as a grey area in the full episode) is presented, including a time-stamped, condensed activity pattern (A = action, C = chat). The dialogue is extracted from the activity log, and the speech bubbles contain excerpts from the coded CRR data that are related to the content of the particular excerpt. (Translated from Finnish). (The full episode is available also in Fig. 10 in Appendix 2)

**Fig. 7:**
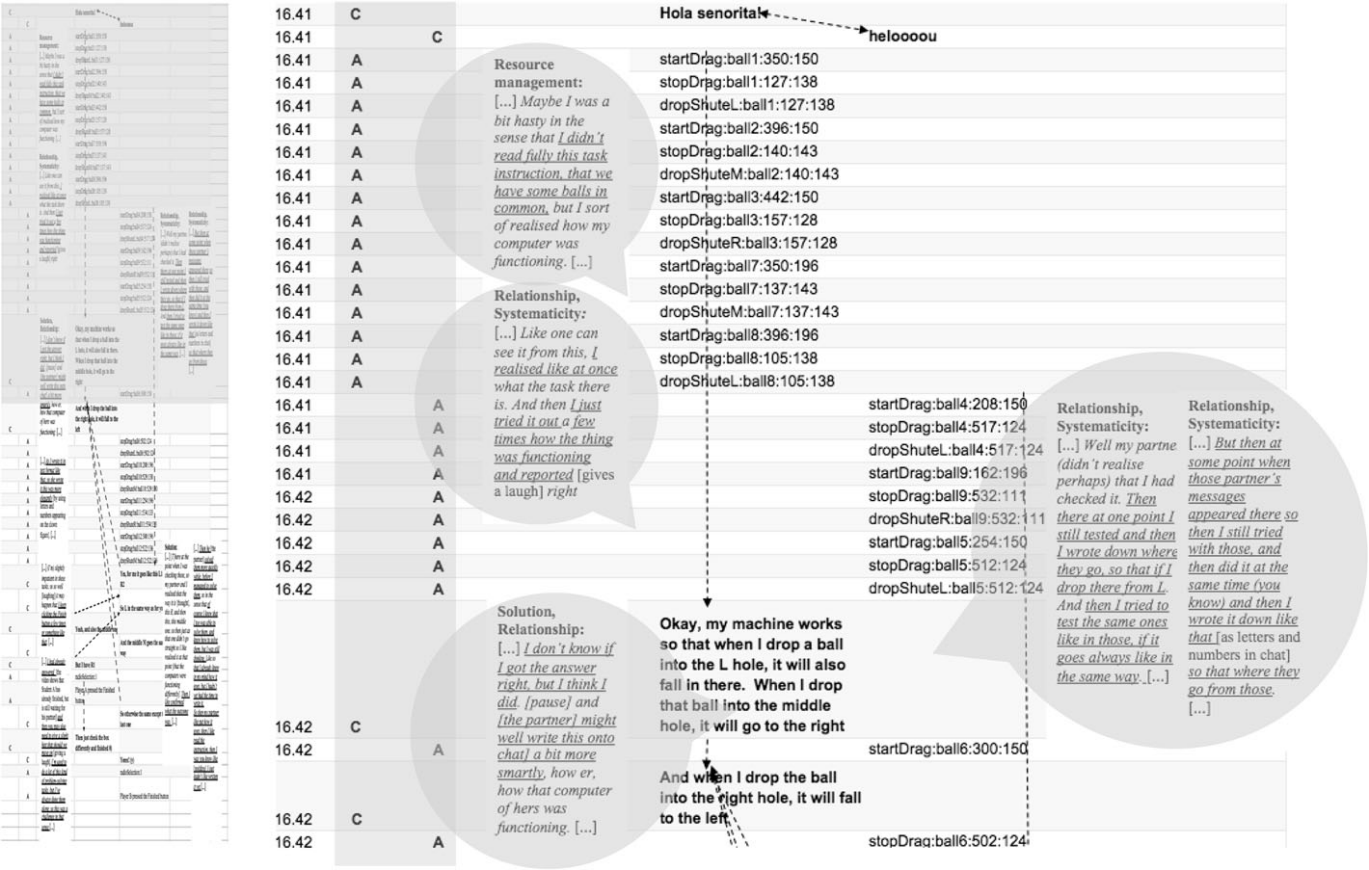
Individual solution endeavour (pair 29, Laughing Clowns). The full episode as a general pattern of interaction is presented on the left. On the right, an excerpt (indicated as a grey area in the full episode) is presented, including a time-stamped, condensed activity pattern (A = action, C = chat). The dialogue is extracted from the activity log, and the speech bubbles contain excerpts from the coded CRR data that are related to the content of the particular excerpt. (Translated from Finnish). (The full episode is available also in Fig. 11 in Appendix 3)

In the symmetric task, Laughing Clowns, the analysis of the process data (activity logs and CRR data) of pairs 24 and 29 confirmed the qualitatively different CPS processes revealed in the directed content analysis (see Table 7). In analysing the interactions, we found evidence of interaction qualities that, for pair 24, revealed a *joint solution endeavour* and, for pair 29, *individual solution endeavours* when completing the task (for micro-interaction patterns, see Figs. 6 and 7; the full episode is available also in Figs. 10 and 11 in Appendixes 2 and 3, respectively). The pattern of pair 24 (joint solution endeavour) depicts an ideal dual-problem space (Barron 2003; see also Alterman and Harsch 2017) where the participants simultaneously focused and developed the content space (cognitive aspects) and the relational space (collaborative aspects). In the content space, they jointly made sense of the significant elements of the task and together reasoned out the task logic, whereas in the relational space, they showed the ability to manage their interpersonal relations as they collaborated (Alterman and Harsch 2017). Pair 29, in turn, showed significant challenges in this respect (individual solution endeavour), which led to a string of low coordination of the activities (Davis et al. 2015) and a self-focused, parallel problem-solving trajectory in the collaborative context (Schneider and Pea 2014; Sinha et al. 2015).

Regarding the social aspects of CPS, in the Laughing Clowns task, the fundamental requirement for successful completion of the task was *interaction* between participants. Participants needed to be aware that their balls were shared and that the most effective way of finding the solution was to assign the balls so that both students would have an adequate and equal opportunity to try their machine and reach a conclusion. In contrast to pair 24, pair 29 did not discuss the allocation but started working independently, dropping and dragging the balls without interacting and coordinating their efforts. Also, evidence of *audience awareness* (i.e. how a participant adapts contributions to increase understanding of others) is missing with pair 29. Students who are proficient in this area would be more likely to interact with their partner between ball drops and adapt their behaviour to best suit their partner’s needs (in contrast, see pair 24). Student 24B stated (see excerpt 1): ‘[…] I understood that she [the partner] has something differently there than on my screen and that I don’t know what she’s got there, so then I felt that I have to explain what I’m seeing […]’ (excerpt 1, student 24B, CRR interview).

In terms of the cognitive aspects of CPS, students with a low proficiency of *resource management* skills may only think of themselves when checking how their machine functions, thereby monopolising the use of resources, which seems to be the situation with pair 29, while more skilled students would more likely recognise the need for shared use of the balls and hence would share them equally (pair 24). Moreover, to reach a solution, the students needed to identify the relationship between the entry and exit point of the balls and determine if there was consistency in how the machines function; the pair needed to construct a way of representing this information and communicating it to each partner, as well as being able to understand the other forms of representation that the partner uses. For example, for pair 29, student A provided a narrative while student B listed pieces of information. Student 29B described their parallel working on the task as follows (excerpt 2):

[…] Well my partner (didn’t realise perhaps) that I had checked it. Then there at one point I still tested and then I wrote down where they go, so that if I drop there from L. And then I tried to test the same ones like in those if it goes always like in the same way. But then at some point when those partner’s messages appeared there, so then I still tried with those, and then did it at the same time (you know) and then I wrote it down like that [as letters and numbers on chat] so that where they go from those […] (excerpt 2, student 29B, CRR interview).

Skilful students will also challenge the patterns and test the assumptions that underpin their observations, which happened with student 29B (excerpt 2). The final step comprised the students comparing their representations so that a decision concerning the similarity of the functioning of the clown machine could be made (*solution*). Pair 24 communicated throughout their activities, whereas pair 29 only discussed their shared understanding toward the end. If the task was not a forced collaboration exercise (thanks to the last concluding question), student 29A would have been able to solve the task quite independently because he also pointed out the following in the CRR interview (excerpt 3):

[…] Like one can see it from this, I realised like at once what the task there is. And then I just tried it out a few times how the thing was functioning and reported [gives a short laugh] right away. I don’t know whether one could have like [a short laugh] built some cooperation there like so that one would first have like told the instruction in the way one understood it […] (excerpt 3, pair 29A, CRR interview).

#### Case 2: balanced versus unbalanced CPS process in terms of the dynamics of interaction observed in the asymmetric task Olive Oil

**Fig. 8:**
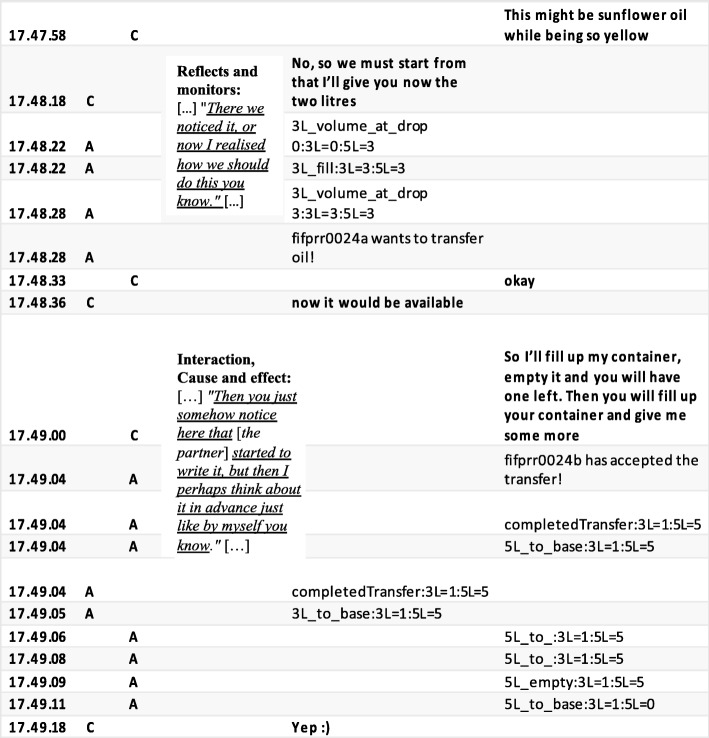
Balanced CPS process in terms of the dynamics of interaction (pair 24, Olive Oil). The excerpt includes a time-stamped, condensed activity pattern (A = action, C = chat); dialogue extracted from the activity log, and the speech bubbles contain excerpts from the coded CRR data. (Translated from Finnish)

**Fig. 9:**
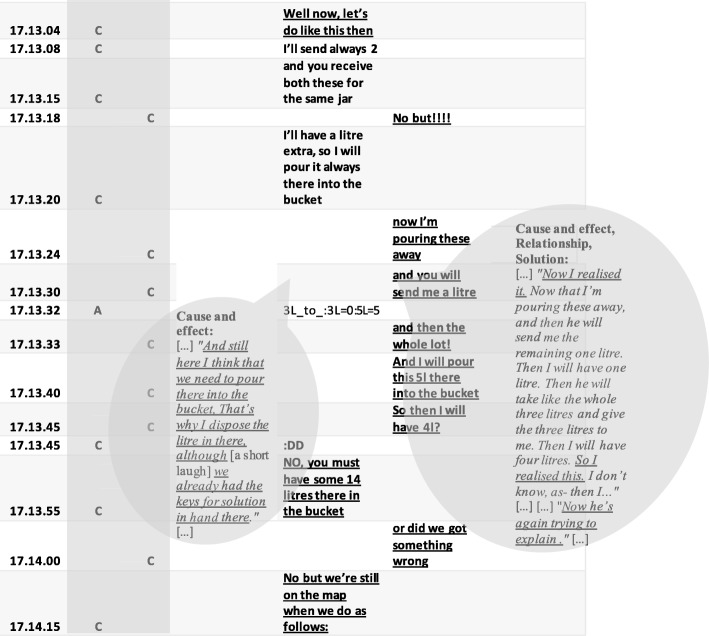
Unbalanced CPS process in terms of the dynamics of interaction (pair 29, Olive Oil). The excerpt includes a time-stamped, condensed activity pattern (A = action, C = chat). The dialogue is extracted from the activity log, and the speech bubbles contain excerpts from the coded CRR data. (Translated from Finnish)

**Fig. 10:**
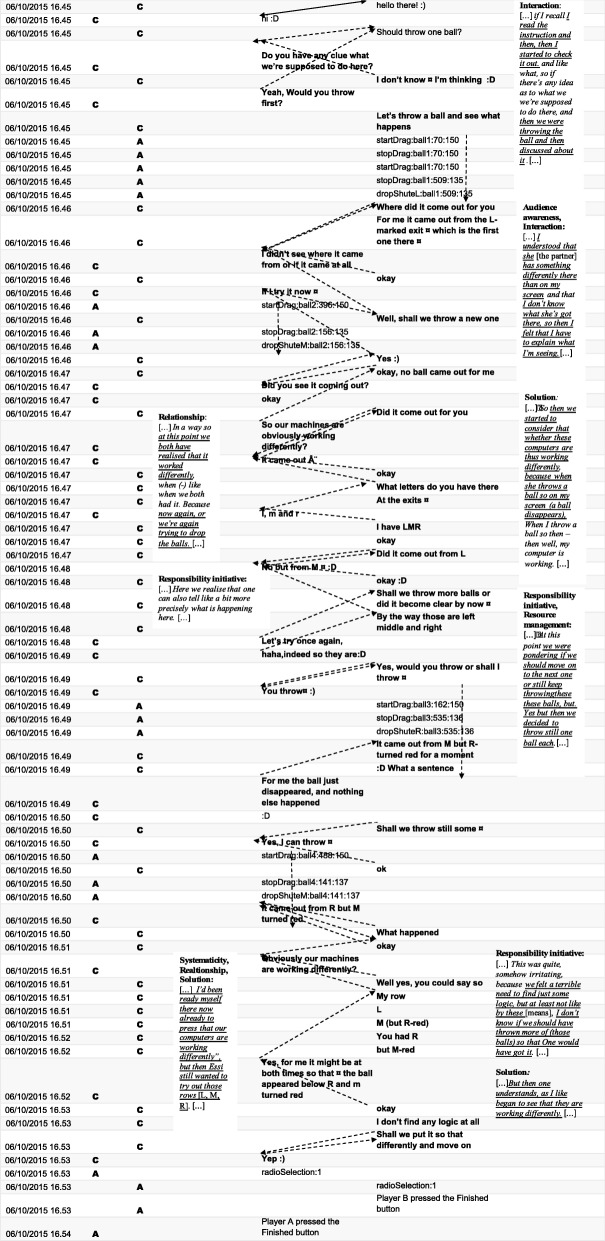
The full episode of pair 24 (joint solution endeavour) in the Laughing Clowns task

**Fig. 11:**
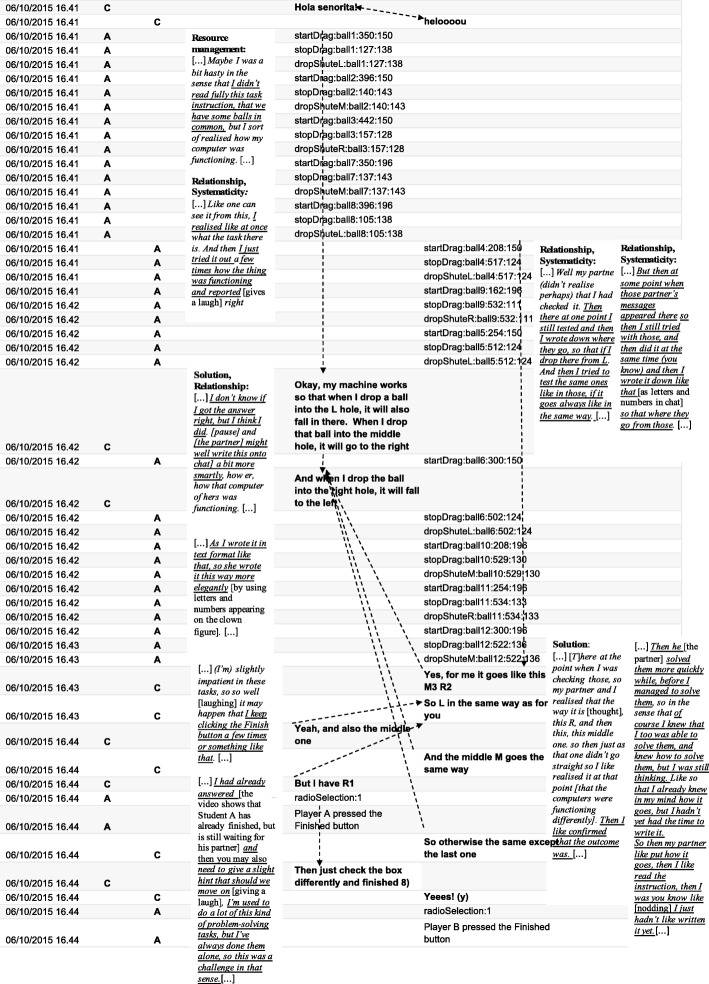
The full episode of pair 29 (individual solution endeavour) in the Laughing Clowns task

In contrast to the Laughing Clowns task, in the asymmetric task Olive Oil, the cumulative accounts obtained from the directed content analysis of pairs 24 and 29 (Table 8) were in line with the task design, even emphasising more social aspects than the original design of the Olive Oil task did. However, when comparing these cumulative accounts from the directed content analysis on the CRR data to the micro-level interaction processes visible in the activity logs, there were evident differences regarding the *quality* of collaboration across the two pairs. Accordingly, the micro-interaction contents revealed qualitatively different CPS processes as *balanced* versus *unbalanced dynamics of interaction* (for micro-interaction patterns, see Figs. 8 and 9). Even though the asymmetry of the task style was designed to force interaction in pairs, the quality of the ways in which the pairs negotiated the task seems to vary. Pair 24 continued to resemble a joint exploration process. Pair 24 negotiated their explorations and jointly developed and discussed their understandings to solve the task (see Fig. 8). In pair 29 (see Fig. 9), the dynamics of the interaction in their problem-solving process were unbalanced in terms of the power relations being unequal positions of status (see e.g. Simpson et al. 2017). Student A tended to assign the role of ‘leader’ or ‘driver’ to himself in the shared activity, assigning student B the role of ‘follower’ or ‘passenger’ (Schneider and Pea 2013; Shaer et al. 2011). However, student B seemed to accept this role. In Figs. 8 and 9, the qualitative different dynamics of the interaction are exemplified. Figure 8 depicts the pattern of pair 24, which can be characterised as a balanced, joint interaction, with equal distribution of work and responsibilities. Figure 9, in turn, shows an example of the unbalanced nature of the dynamics of the interaction in pair 29. Figure 9 gives an example of an explicit disagreement (Richie 2002; Simpson et al. 2017) in which student 29A shortly questions his partner’s proposal of the possible steps to reach the problem solution, favouring his own idea. Figure 9 displays the pivotal moment for pair 29 when the idea of how to solve the task is crystallised for student B. However, the idea is rejected by student A, who simply continues to implement his own idea of how to reach the solution. When student B seemingly asked for confirmation, ‘[…] or did we got something wrong […]’, student A merely replied using a delicate imperative: ‘[…] No, but we’re still on the map when we do as follows: […]’.

Based on the asymmetrical task design, at the beginning of the Olive Oil task, student B depended on the actions and interaction of student A. Student A, even though highly active, did not truly interact with student B during the problem-solving efforts; the student also failed to respond to the co-student’s efforts to solve the task. Instead, student A attempted to direct the process, as is acknowledged by his partner in the CRR interview (excerpt 4): ‘[…] I just thought as my partner told me there like quite a lot what I have to do, so I thought then that he like figures this out […]’ (excerpt 4, student 29B, CRR interview).

From the cognitive aspects of CPS, *problem analysis* is the first step in planning. Problem analysis requires participants to explore the task and define subtasks and construct subgoals to successfully solve the problem together. In pair 29, even though student A seems focused on unravelling the task, the subgoals set were not aligned at the pair level. Also, before organising the necessary steps to solve the problem, the students needed to share information and explain the resources available, as was efficiently discussed by pair 24. Pair 29 did share information but did not systematically discuss the elements each had on his or her screen, as was the case with pair 24. This caused misunderstandings. Student 29A reflected on their working (excerpt 5): ‘[…] We went through what we have there. [Pause] What we can see on our screen, but actually we went through this only later […]’ (excerpt 5, student 29A, CRR interview).

The problem analysis phase is followed by organising the necessary steps to solve the task. *Cause and effect* required the students to successfully think of steps ahead of their current problem state and work out subtasks before acting. In the Olive Oil task, the essential requirement was, in advance, to work out sequences of movements to achieve the goal, that is, to fill the jar with 4 l of oil. Pair 24B reflected on this (see excerpt 6): ‘[…] I like this kind of things where you must think like, those few moves so that you’ll get them, you reach the final result […]’ (excerpt 6, student 24B, CRR interview).

As the circumstances change, proficient students were better able to adapt their reasoning and re-organise their understanding of the task at hand (i.e. *reflects and monitors*; Hesse et al. 2015). For example, pair 24 recalled the following (excerpts 7 and 8): student A said, ‘[…] So then I was still thinking that if one could pour it from the bucket, so I realised (that nothing is coming from there). There we noticed it, or now I realised how we should do this you know […]’ (excerpt 7, student 24A, CRR interview). Student B, in turn, formulated their reasoning as follows:

[…] Well then there was the thing that it would have gone there into the bucket the right amount, that is four litres, but then we started to think that perhaps it’s such a glass container after all, where we should have it. [Pause, the video shows that 24b has a full glass container.] There is now that one litre there in [partner’s] bucket. Now, she only should three more, so then there would be the four litres in the bucket. But then we realise that it ought to be a glass container. (We have thought it) wrong […] (excerpt 8, student 24B, CRR interview).

In the Olive Oil task, the cognitive element, *relationship*, was visible in the chat because student A or B recognised and communicated the significant details of what follows if the jar contains only 1 l of oil. In pair 29, already in the middle of the entire problem-solving process, student B came up with a correct, detailed plan of the sequences of movements to be executed to achieve the desired goal state, but student A did not recognise this effort, continuing with his ongoing actions, so the idea was dismissed (Fig. 7). Student 29B depicted the following (excerpt 9):

[…] As I know that it [own idea] is right, and then as he was yet that ‘let’s do like this’ so then I began to think that which one the container actually is. And as he seemed to be so certain there in his explanation. And I was certain as well, but as he was like that ‘let’s do like this […] (excerpt 9, student 29B, CRR interview).

Later on, student 29A came up with the same idea, as student 29B stated (see excerpt 10):

[…] Now he would have liked to do it like that, then [laughter]. Now he’s explaining the same what I explained to him already earlier [a short laugh]. As now he tells ‘I’ll send you 1 and then I’ll send 3’ so there it was. And I was like in fact I told you so! [a short laugh]! I knew this all right, that then it will be solved. That I knew it all the time. I knew this before him […] (excerpt 10, student 29B, CRR interview).

## Discussion and conclusions

The aim of the current exploratory case study was to examine the micro-interactions in pairs during the online assessment of CPS skills in the ATC21S assessment environment. The present study shifted from an individual and solution-orientation focus on the CPS process to the group-relational aspects of CPS. Our grounding assumptions were based on the definition of the CPS construct as comprising multiple, interacting subelements or skills rather than a linear set of processes (see Care et al. 2016; Hesse et al. 2015; Scoular et al. 2017). In the current study, we asked how student pairs accomplished ambiguous CPS tasks and how their negotiations toward shared understanding were manifested in micro-interactions during the CPS process. We were interested in whether different sets of interactions can underlie successful completion and whether the nature of a task stimulates different sets of interactions. Our case-based portraits showed that despite the students’ comparable and relatively high or moderate CPS performance outcome scores, especially in social skills (scored between the levels of 5–6, with 6 being the highest skill level), variations in micro-interactions occurred across pairs, for example as individual and joint solution endeavours and as balanced and unbalanced CPS processes in pairs. Also, the asymmetrical task type, in comparison to the symmetrical task type, appeared to be more effective in forcing interactions in pairs. However, this did not guarantee the actual quality of the micro-interactions.

Accordingly, in small-group collaboration, there are multiple interacting elements that contribute to the structure and flow of collaboration, such as the aforementioned elements that are related to the joint problem space (Alterman and Harsch 2017; Roschelle and Teasley 1995). As was also witnessed in case 1 (Laughing Clowns), significant challenges in collaboration may arise if participants do not attempt to coordinate their individual perspectives in the joint problem space. Participants who sensed a co-presence perceived their partners and paid attention to each other (Alterman and Harsch 2017). As shown (e.g. Goffman 1963), to achieve a sense of co-presence is essential for high-quality collaboration. This is, however, more demanding when students are operating in online settings, such as the context of the current study.

In CPS, the process of constructing and maintaining a shared understanding of the task at hand is needed for collaborative learning to evolve (Dillenbourg et al. 2016; Roschelle and Teasley 1995). Building a shared understanding is highly contextual: the media may facilitate or hamper interactions. When compared with co-located collaboration, in an online setting, the creation of a shared understanding requires additional effort from the participants (Dillenbourg et al. 2016) because to regulate interactions and achieve agreement is much more complex (Alterman and Harsch 2017). Here, there may be pressure to achieve joint goals with reduced levels of sharing. But as Davis et al. (2015) stated, normally, in joint activity, people bring their own, individual goals to the situation, but at the same time, they jointly work toward a definition of the situation that works to give the interaction coherence (p. 61). In case 2 (Olive Oil), the roles taken and assigned (i.e. student A as a leader, student B as a follower, see Schneider and Pea 2013) may reflect (following Simpson et al. 2017) the ways in which power relations and intellectual authority are negotiated in group interactions in CSCL settings. Despite the many positive promises of CSCL, such as increased learning gains resulting from joint interaction, Simpson et al. (2017) noted the dangers of CSCL environments, such as the possibility of hampering the learning opportunities of those students who lack a voice and are subjected to their co-students’ instructions (see also Langer-Osuna 2016).

Consequently, as Dillenbourg et al. (2016) pointed out about collaborative learning in general, the tasks are primarily designed to facilitate shared meaning and collaboration. In the CPS construct examined here, the tasks, even though being primarily designed to measure individual student’s CPS skills in a collaborative situation, cannot be successfully completed without the pair (e.g. Care et al. 2015, 2016; Hesse et al. 2015; Scoular et al. 2017). However, collaborative activity and learning should not be considered something that would automatically happen: despite the similar tasks and equal support provided for the participants, the pedagogical designs can be perceived and interpreted differently by different participants (e.g. Arvaja and Pöysä-Tarhonen 2013; Simpson et al. 2017; Sinha et al. 2015). For example, the degree of shared understanding that the pairs needed to reach was related to the type of task they needed to perform (Dillenbourg et al. 2016). In case 1, the symmetry of the resources available for the pairs might have diluted the effect of interdependence. In case 2 (Olive Oil), in turn, the asymmetry of the task activated positive interaction patterns, such as shared negotiations and true acts of collaboration, as expected from this style of task to ensure the removal of, but not guaranteeing, unbalanced interactions. In line with the findings of Simpson et al. (2017), this seemingly influenced the performance and productivity of pair 29.

Taken together, successful collaborative problem solving is a complex combination of multiple intertwined factors, such as cognitive, social and emotional factors (Ludvigsen 2016). Here, to better understand how participants produce learning in collaborative situations requires a focus not only on how they build shared meaning but also on how they engage in this activity (Dillenbourg et al. 2016; Stahl 2007). Engagement may appear at various levels, that is, at the behavioural level (i.e. effort and contributing to the task), social level (i.e. the quality of socio-emotional interaction and equitable participation) and cognitive level (i.e. planning, monitoring and evaluation) (e.g. Sinha et al. 2015). In relatively limited collaborative situations, such as in case 1 (Laughing Clowns), a rather shallow interactional quality might be enough to complete the relatively simple and short task of a symmetric style. However, in the other tasks studied, being richer and asymmetrical in their designs, such as case 2 (Olive Oil), the other participant’s resources and understanding become more critical. But in our data, patterns of interactional difficulties or even breakdowns could be found with the same pair that had difficulties in task 1 (Laughing Clowns) but because of the increasing complexity, not in such linear modes as presented here. In terms of successful task completion, when troubles arise in the students’ interaction, how pairs are able and motivated to repair these encounters is crucial.

Furthermore, deepening our understanding of the appearance collaborative aspects of CPS during an online assessment of CPS skills, the current study contributes to the methods of analysis to help visualise the interaction patterns within a pair to reveal their collaborative approach (or lack of). We used a case study method (see e.g. Baškarada 2014; Gerring 2004) to carefully observe student pairs building shared meaning and acquiring CPS practices in an online assessment context. We applied multiple data sources and phases of analysis and, in this way, succeeded in maintaining a chain of evidence for the audience to follow (see Baškarada 2014; Yin 2009). Also, the methods used were rich in the sense that they combined direct evidence due to an objective process data but were combined and seen in relation to the participants’ subjective descriptions, as cued by the retrospective reporting (CRR) data. This can be seen to enhance the validity of the current study and reduce its bias (see Baškarada 2014). Also, our findings reinforce the recent understanding that to better grasp the quality of social interaction, one must extend the understanding from cumulative accounts of interaction toward grasping the temporality in the data (e.g. Kapur 2011; Kapur et al. 2008; Reimann 2009). Taken together, the depth of the analysis is the primary strength of the case study method (Gerring 2004). As a case study, the aim is not to generalise to populations but rather to arrive at conceptualising the regularities of small-group processes pertaining to the CPS construct (see also Stahl 2017) and to transform tacit knowledge into explicit knowledge (Baškarada 2014). The Laughing Clowns case illustrates a quality contrast of micro-interactions in the most condensed and continuous form, as opposed to the micro-interactions in the other tasks of the bundle students comprised, such as Olive Oil, which required more comprehensive extrapolation. The examples chosen here, however, seem to be representatives of the general quality of the data corpus produced by these two pairs across all the tasks of the bundle.

Finally, because our exploratory case study was conducted in a highly structured assessment environment online, one characterised by a challenging communication channel (chat) and with pairs as the unit of analysis, to be able to generalise the contrasting cases beyond this special research design requires replication with more subjects and additional case examples. Also, it should be noted that it is unclear whether and how the assessment situation, even with it being voluntary, impacted the ways in which the students collaborated. Further, because a CPS activity is associated with both task and social regulation, one of the future lines of investigation in this context could be whether students operating in an online setting may benefit from tools and support structures to enhance regulation activities and awareness between them. For example, to include novel methods, such as dual-eye tracking when working on online collaborative tasks analysed in respect to identified key interactional events or focusing on the ‘micro-monitoring’ of a partner’s behaviour in dyads (Schneider and Pea 2013), could enrich the understanding of CPS. Moreover, in the current study, the challenges discovered in terms of collaboration were not particularly new and pertained to the relatively common practical ills of group work in varied contexts of study (i.e. Barron 2003; Simpson et al. 2017; Sinha et al. 2015). Yet, our case-based approach, with its multiple sources of data and phases of analysis that carefully displayed the processes of pairs building acquired CPS practices, is still rare, even in the area of collaborative learning (Schwarz and Baker 2017; Stahl 2017). Therefore, in line with enriching the research debate on CSCL called for by Law et al. (2017), by analysing temporal interaction processes in dyads, for example, by identifying special problems in the appropriation and use of assessment practices online, this process-orientated approach with its micro-level perspective has the full potential to inform pedagogical design and refinement of practices in this respect.

## Appendix 1

**Table 9:** Examples of the coded primary data from the cued retrospective reporting (CRR) data (from the Laughing Clowns task)

Skill/element	Behaviour indicator (as assessed in the ATC21S environment)	An example of the CRR data captured for coding the category in the Laughing Clowns task
Interaction	Interacting with partner (e.g. presence of chat before allowing partner to make a move)	‘But then she [the partner] was again so much smarter here, that she like started to explain what she was seeing.’ ‘In this task it felt surprisingly difficult just to think that one should always explain to the other person what one is seeing.’ ‘If I recall I read the instruction and then, then I started to check it out. and like what, so if there’s any idea as to what we we’re supposed to do there, and then we were throwing the ball and then discussed about it [a short laugh]’ ‘Here one somehow started to be like, how would I put it, you know like (one immediately started to tell what there is and where) and describe it like all the time asking that what do you see, like to tell what is happening on your own screen’.
Audience awareness	Adapts contributions to increase understanding for partner	‘I understood that [the partner] has something differently there than on my screen and that I don’t know what she’s got there, so then I felt that I have to explain what I’m seeing. And I like wanted to ask what – then we threw [the partner threw] the ball again [a short laugh] and I had this same that I couldn’t see at all what was happening, and I see that the ball disappears.’ ‘As I have now put, (is there) I think I too, [the partner] too got. After this I might put again for M3, no I mean, what did I get there? So yes, I got R3. No, it was R2. Yes. Then here I was really thinking how it might, or in fact I was thinking whether it would go somehow like in a logical order, so that for me for instance some M3 is always repeated, then there will be R2, some certain [patterns] like these. And then after this, now soon here, I think, [the partner] will like tell how these go, you know. Then I am like aha, yes, right, yes and then, but she just gets always R, did she get always R1 then and for me R2 (or somehow like that).’ ‘I think [the partner] did tell at some point that they go. Because she’s [told] me, so she told that they go, how the clown’s head goes. Then I just thought that well, should we still make sure, so that let’s use all those balls.’
Responsibility initiative	Takes responsibility for progress for the group task	‘At this point we were pondering if we should move on to the next one or still keep throwing these balls, but. Yes but then we decided to throw still one ball each.’ ‘I tried as quickly as possible so that we’ll get this done.’ ‘And then we regarded that we should move on. (This was indeed a difficult one.)’
Resource management	Manages resources	‘At that point I was a little afraid that did she realise that the balls ran out.’ ‘Perhaps I was a bit in a hurry there. [A short laugh] and there we noticed that they [the balls] were actually jointly for us. I think we didn’t read it quite properly the instruction from there. As I was putting this ball into the [clown’s] mouth but then it suddenly disappeared as my partner had put it.’ ‘And then we didn’t realise [giving a laugh] that they are the same balls. So in principle as I have two balls left, then she [thought] that she, too, has. [Pause] So then we just realised that they are jointly for us.’
Systematicity	Implements possible solutions to a problem	‘I like in a way tried, so that I’d been ready myself there now already to press that our computers are working differently, but then [the partner] still wanted to try out those rows [L, M, R]. Uses half of the balls to cover the positions in a sequential order.’ ‘Well, we both had noticed that if you dropped a ball when the mouth is there at M, so it will then go to number three. And then we decided to test those other letters as well. As we had to find out whether the machines were similar or not.’ ‘Here you could perhaps see how different I and [the partner] are in a way, because I would have been at this stage [points to the chat screen] sort of ready in principle like to click that we are ready. And then [the partner] like wanted that I would test it with two more balls, although I had already noticed at this stage that okay, it works this way, from M to 2 and from R to 1, you know, so I don’t need to test it any further. So [the partner] thought that I should still with two more balls, although I would have been like ready at this point. [Pause, on the video, she is browsing upwards on the chat screen.] So there I just checked that [the partner] had also earlier, no I mean what she had, earlier something, I think she tested still one something. It appeared there like in the same way as what she had got there earlier. So we could see that it was like.’
Relationships	Identifies connections and patterns between elements of knowledge	‘In a way so at this point we both have realised that it worked differently, when (-) like when we both had it. Because now again, or we’re again trying to drop the balls. The two students come to an agreement on how their machine works.’ ‘So then we started to consider that whether these computers are thus working differently, because when [the partner] throws a ball so on my screen (a ball disappears), When I throw a ball so then – then well, my computer is working, (but). We considered that the screens are anyway quite identical, there are those left, middle and right, (-- they are the same). [Pause] I can’t recall what I was thinking there [giving a laugh] but probably that where is it coming out [the ball]. and when it came out from a different point, so (I thought they were working) differently, because if you throw that ball in there and it comes at once, (Or out I mean). (But then it could be nicer you know also to look a bit that which ball.)’ ‘So I started to pay attention to whatever is happening then, launching those balls. [Pause] And I move – I launched this one ball and then I realised, it made the R. I think I tried again.’
Solution	Correct answer	‘Then just as that one didn’t go straight so I like realised it at that point [that the computers were functioning differently]. Then I like confirmed what the outcome was Selection of the correct option by Students A and B on how their machines work.’ ‘(we noticed there that) they’re really working differently, as [the partner] had got those other letters.’ ‘I’d been ready myself there now already to press that our computers are working differently.’

The categorisation matrix of the directed content analysis applied the same behavioural indicators that were used in the automated coding procedures in the ATC21S environment (see Care et al. 2015)

## Abbreviations

ATC21S: Assessment and Teaching of 21st Century Skills
CPS: Collaborative problem-solving
CRR: Cued retrospective reporting
CSCL: Computer-supported collaborative learning
